# mTORtuous effect on the elastic heart

**DOI:** 10.18632/oncotarget.4788

**Published:** 2015-07-08

**Authors:** Jonathan C. Schisler, Rosalind A. Coleman

**Affiliations:** McAllister Heart Institute, The University of North Carolina at Chapel Hill, Chapel Hill, NC, USA

Both during normal physiological development as well as in the face of cardiac stress, the heart exhibits enormous plasticity in growth, remodeling, and atrophy. Central to this adaptive ability is the heart's metabolic capacity to react quickly both anabolically and catabolically in order to maintain cardiac output. In response to hemodynamic stress, the heart adapts to maintain overall contractile function via pathological remodeling that often includes cardiomyocyte hypertrophy as well as changes in cardiomyocyte metabolism that eventually lead to contractile dysfunction and heart failure. It is unclear as to whether the metabolic changes that accompany pathological hypertrophy are adaptive or maladaptive; however, as in most complex biological systems, the answer likely resides somewhere in the middle. Goodwin et al. demonstrated in 1998 that hemodynamic stress increases carbohydrate use for ATP generation and induces cardiac remodeling [1]. Numerous subsequent studies have attributed the change in metabolism and cardiac remodeling to activation of the mammalian (or mechanistic) target of rapamycin (mTOR), a kinase that is a primary regulator of myocardial protein synthesis. Recent experimental models that manipulate cardiac metabolism and mTOR signaling have provided new insights into the relationship between metabolic and structural remodeling (reviewed in detail by Kundu et al. [2]). One hopes that such models will identify metabolic changes that precede remodeling in order to find therapeutic targets for cardiac disease.

We developed a mouse model of heart-specific inactivation of ACSL1, a membrane-associated enzyme present on the mitochondria and endoplasmic reticulum that catalyzes the activation of long-chain fatty acids to form acyl-CoAs [3]. The ACSL1 isoform appears to deliver newly formed acyl-CoAs to carnitine palmitoyltransferase, thereby targeting them for mitochondrial β-oxidation. As a result of ASCL1 inactivation, fatty acids can no longer be oxidized to contribute to ATP synthesis, and hearts undergo a dramatic switch to glucose metabolism as their primary source of energy [3]. Although *Acsl1^H−/−^* mice develop mTOR-dependent cardiac hypertrophy, surprisingly, the cardiac function of *Acsl1^H−/−^* mice remains resilient. After 20 weeks of ACSL1 inactivation, systolic function remains unaffected [3] even in the presence of early stage diastolic dysfunction [4]. Systolic heart failure is commonly observed in other models in which mTOR activity is increased, more specifically mTORC1 [5], or when reliance on glucose metabolism is forced (for examples see Schisler et al. [6]). Because *Acsl1^H−/−^* mice do not develop systolic heart failure, our mice provide a useful model to identify genes and metabolites that may contribute specifically to diastolic dysfunction or to the maintenance of systolic function and decreased cardiac mortality even when mTOR is markedly activated.

First, our data [6] support recent studies demonstrating that cardiac metabolic remodeling precedes the hypertrophic response. In an unbiased approach, we observed multiple glycolytic metabolites such as glucose 6-phosphate, mannose 6-phosphate, and fructose 6-phosphate that are likely to contribute to the activation of mTOR. Additionally, metabolomics profiling of *Acsl1^H−/−^* hearts also revealed a surprising increase in 13 metabolites related to cysteine and glutathione metabolism. We hypothesize that the upregulation of glutathione metabolism may be a response to a selective, glutathione-restricted oxidative stress observed after 10 weeks of ACSL1 inactivation. Dietary supplementation can modulate the cysteine-glutathione pathway in animal models [7] and *in vivo* studies that modify this pathway should provide further insight into whether cysteine-glutathione metabolism is, in fact, cardioprotective when mTOR is activated. In addition, we identified the up-regulation of several starvation-related atrophy genes that contain amino acid response elements, as well as multiple genes associated with amino acid deprivation. Although the increased expression of these genes, including the atrogene *MuRF1*, are usually associated with atrophy and loss of skeletal muscle mass, we may be observing an unappreciated component of cardiac metabolic reprogramming that uses selective protein catabolism to maintain cardiac output, undeterred by mTORC1-induced protein synthesis. Furthermore, the finding that mTORC1-inhibited autophagy is prominent in *Acsl1H^−/−^* hearts (Grevengoed and Coleman et al., unpublished observations), suggests not only alternative mechanisms of protein degradation, but also highlights the preserved systolic function despite structurally abnormal mitochondria with diminished respiratory function.

Supported by DK59935 (to R.A.C) and GM061728 (to J.C.S.).
